# An ERP study on proactive and reactive response inhibition in individuals with schizotypy

**DOI:** 10.1038/s41598-021-87735-5

**Published:** 2021-04-16

**Authors:** Lu-xia Jia, Xiao-jing Qin, Ji-fang Cui, Qi Zheng, Tian-xiao Yang, Ya Wang, Raymond C. K. Chan

**Affiliations:** 1 Neuropsychology and Applied Cognitive Neuroscience Laboratory, CAS Key Laboratory of Mental Health, Institute of Psychology, Beijing, China; 2 Department of Psychology, University of Chinese Academy of Sciences, Beijing, China; 3 Research Center for Information and Statistics, National Institute of Education Sciences, Beijing, China

**Keywords:** Cognitive neuroscience, Human behaviour

## Abstract

Schizotypy, a subclinical group at risk for schizophrenia, has been found to show impairments in response inhibition. However, it remains unclear whether this impairment is accompanied by outright stopping (reactive inhibition) or preparation for stopping (proactive inhibition). We recruited 20 schizotypy and 24 non-schizotypy individuals to perform a modified stop-signal task with electroencephalographic (EEG) data recorded. This task consists of three conditions based on the probability of stop signal: 0% (no stop trials, only go trials), 17% (17% stop trials), and 33% (33% stop trials), the conditions were indicated by the colour of go stimuli. For proactive inhibition (go trials), individuals with schizotypy exhibited significantly lesser increase in go response time (RT) as the stop signal probability increasing compared to non-schizotypy individuals. Individuals with schizotypy also exhibited significantly increased N1 amplitude on all levels of stop signal probability and increased P3 amplitude in the 17% stop condition compared with non-schizotypy individuals. For reactive inhibition (stop trials), individuals with schizotypy exhibited significantly longer stop signal reaction time (SSRT) in both 17% and 33% stop conditions and smaller N2 amplitude on stop trials in the 17% stop condition than non-schizotypy individuals. These findings suggest that individuals with schizotypy were impaired in both proactive and reactive response inhibition at behavioural and neural levels.

## Introduction

Response inhibition refers to the ability to withhold either dominant response tendency or already-activated responses^1^, which is a core component of executive control^2,3^. Deficit in response inhibition has been found in individuals with schizophrenia spectrum disorders^1,4,5^. In patients with schizophrenia, response inhibition impairment was not only associated with behavioural problems including impulsivity^6,7^ and aggression^8^ but also correlated with clinical symptoms. For example, abnormal prefrontal activity during response inhibition was associated with excitement/impulsive symptoms^9^. In individuals with schizotypy, response inhibition impairment was correlated with interpersonal and disorganized schizotypal traits^5^. Therefore, investigating response inhibition is an important perspective to understand the cognitive impairments and behavioural disturbances in schizophrenia spectrum disorders.

A key paradigm to assess response inhibition is the stop-signal task^1,10,11^. In the stop-signal task, participants are instructed to respond to “Go” stimuli, but to stop the response if the stimulus was followed by a “Stop” signal. The time taken to withhold the “Go” response when the stop signal occurs (i.e., the stop signal reaction time, SSRT) is an indicator of response inhibition. Studies have shown that patients with schizophrenia were impaired in response inhibition, showing longer SSRT at the behavioural level, and such impairment was correlated with abnormal activations of striatal cortex, prefrontal cortex and related connections^12,13^. Longer SSRT has also been found in individuals with schizotypy^4,5^ and children at genetic risk for schizophrenia^14^. These findings provide preliminary evidence that impairment of response inhibition may be a biological marker for schizophrenia^15^. However, few studies have examined the neural mechanisms underlying impaired response inhibition in individuals at risk for schizophrenia^16^.

The above studies have focused on reactive inhibition, which refers to the cancellation of the planned response at the moment the stop signal occurs. However, a host of prior studies have suggested that response inhibition could be divided into two forms: reactive inhibition and proactive inhibition^17–20^. Proactive inhibition entails the anticipation and preparation to inhibit forthcoming actions when necessary^21–23^. Anticipating the appearance of a future stimulus is important for goal-directed behavior^24,25^. For response inhibition, response is slowed in “Go” trials when anticipating a stop signal (proactive inhibition) compared with a baseline condition that no stop signals are anticipated^10^.

A majority of previous studies have shown that schizophrenia patients exhibited impaired proactive inhibition compared with healthy controls^13,26,27^, suggested by a smaller increase in response time in “go” trials with the increasing probability of the stop signal. Moreover, failure to activate relevant brain regions (e.g., the right striatum, the right inferior frontal cortex, and the left and right temporo-parietal junction) and reduced cortico–cortico/intracortical connections may also serve as an evidence for proactive inhibition impairment in schizophrenia^26,27^. Barch and Ceaser found that proactive control impairment in schizophrenia was a common mechanism driving their deficits in the cognitive domain, such as working memory, executive control, and episodic memory. For example, schizophrenia patients with the poorest proactive inhibition also exhibited the shortest working memory span^26^. It is agreed that proactive inhibition processes reflect a sensitive marker of schizophrenia^26,28,29^. Moreover, a smaller increase in response time of “go” trials as the stop probability increasing has also been observed in siblings of schizophrenia patients compared with healthy controls^26^.

Individuals with schizotypy has been considered to be at-risk individuals for schizophrenia^30^, sharing a range of characteristics similar to schizophrenia^31^, e.g., genetic^32^, neuroimaging^33^, and neuropsychological^34^ characteristics. A better understanding of schizophrenia can be achieved by studying this population through avoiding confounding factors such as medication and chronicity^35^. Preliminary findings have also suggested that individuals with schizotypy have also exhibited longer SSRT, i.e., impaired reactive inhibition^4,5^. However, the neural mechanism underlying it has yet been studied. Moreover, no studies have examined proactive inhibition in individual with schizotypy.

In the present study, an adapted stop signal task was administered with electroencephalography (EEG) recording^22,23^, in which stop-signal probability was manipulated, allowing us examine whether proactive and reactive inhibition would be altered in individuals with schizotypy at behavioural and neural levels. EEG is a neurophysiological tool with a high temporal resolution of milliseconds, which is usually used to examine the temporal course of neural modulation of cognitive processes. For proactive inhibition, we focused on P3 component elicited by go trials. The centro-parietal positivity P3 peaks at approximately 300–600 ms after stimulus onset^36^, which is associated with inhibitory processing. For example, a study using Flanker task showed that incongruent trials exhibited larger P3 compared with congruent trials^37^. The amplitude of P3 is sensitive to the amount of attention resources. In this study we also examined N1 component elicited by go trials, N1 is an early (50–250 ms after stimulus onset) component and is associated with sensory processing and stimulus discrimination^38,39^. Moreover, the amplitude of N1 can be influenced by attention, and increased attentional resources are reflected as greater N1 amplitude^40^. For reactive inhibition, we focused on N2 evoked by stop trials^41,42^. N2 generates from prefrontal cortex and peaks at around 200–250 ms after stimuli onset^43–45^. Usually the amplitude of N2 was larger for failed versus successful stopping, indicating that N2 reflects error monitoring and detection processes^2,46^. Patients with schizophrenia exhibited reduced N2 amplitude for failed stop trials compared with healthy control individuals^47^, indicating impaired ability in response monitoring.

Taken together, the present study aimed to examine whether individuals with schizotypy would exhibit impairment in proactive and reactive response inhibition in an adapted stop signal task, and whether they would exhibit abnormalities in event related potential (ERP) components associated with these processes. Given proactive inhibition is impaired in patients with schizophrenia and their non-psychotic siblings^13,26^, we hypothesized that proactive inhibition would also be impaired in individuals with schizotypy. Specifically, we expected that schizotypy individuals would exhibit a lesser increased response time in go trials while stop trials are anticipated than in go trials while stop trials are not anticipated and altered P3 and N1 amplitude in go trials compared with non-schizotypy individuals. Given that previous findings have demonstrated that schizotypy individuals were impaired in reactive inhibition^4,5^, we also hypothesized that reactive response inhibition would be impaired in schizotypy individuals at both the behavioural and ERP levels, reflecting as longer SSRT and smaller N2 amplitude to “stop” trials compared with non-schizotypy individuals.

## Results

### Sample characteristics

For non-schizotypy group, there were 24 participants (5 males, 19 females) and the mean SPQ score was 14.08 (SD = 7.38). For schizotypy group, there were 20 participants (6 males, 14 females) and the mean SPQ score was 45.50 (SD = 9.53). The mean age of the non-schizotypy and schizotypy groups were 22.00 years (SD = 2.06) and 21.45 years (SD = 1.76), respectively. The mean length of education for non-schizotypy and schizotypy groups were 15.42 years (SD = 1.93) and 15.10 years (SD = 1.59), respectively. The two groups did not differ in gender ratio (χ^2^(1) = 0.49, *p* = 0.484), age (*t*(42) = 0.94, *p* = 0.353) and length of education (*t*(42) = 0.58, *p* = 0.561).

### Behavioural results

#### Proactive inhibition (go trials)

**Figure 1:**
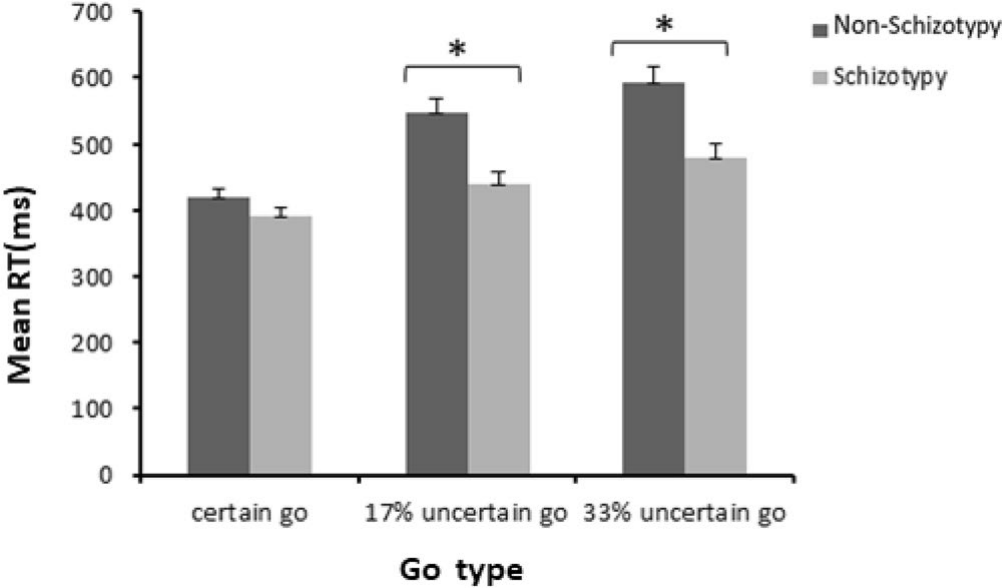
Response time of correct Go trials across different Go type for the non-schizotypy and schizotypy group. *Significant (*p* < 0.05) group differences in post hoc analyses. Error bars represent standard error.

Table 1 summarizes the performances of schizotypy and non-schizotypy individuals. There was a significant main effect of Group (*F*_(1, 42)_ = 13.07, *p* = 0.001, *η*_*p*_^2^ = 0.237), indicating that response time of go trials was longer for non-schizotypy than schizotypy group. The main effect of Go type was also significant (*F*_(2, 84)_ = 64.37, *p* < 0.001, *η*_*p*_^2^ = 0.605), indicating response time of “go” trials increased as the probability of stop-signal increasing. Post-hoc tests (Bonforroni corrected) revealed that the response time of “33% uncertain go” trials was longer than the “17% uncertain go” trials (*p* < 0.001), which was longer than the “certain go” trials (*p* < 0.001). Moreover, the Group × Go type interaction was significant (*F*_(2, 84)_ = 8.06, *p* = 0.004, *η*_*p*_^2^ = 0.161). Simple effect analyses indicated that the non-schizotypy group exhibited significantly longer response time (*M* = 547 ms) than schizotypy group (*M* = 440 ms) in the “17% uncertain go” condition (*F*_(1, 42)_ = 14.08, *p* = 0.001, *η*_*p*_^2^ = 0.251) and the “33% uncertain go” condition (*M*_non-schizotypy_ = 593 ms, *M*_schizotypy_ = 480 ms, *F*_(1, 42)_ = 12.48, *p* = 0.001, *η*_*p*_^2^ = 0.229), but not in the “certain go” condition (*M*_non-schizotypy_ = 420 ms, *M*_schizotypy_ = 392 ms, *F*_(1, 42)_ = 2.83, *p* = 0.10, *η*_*p*_^2^ = 0.063) (Fig. 1). These results suggest a smaller increase in response time with the increased stop-signal probability in the schizotypy relative to the non-schizotypy group.

**Table 1:** Behavioural results on stop signal task [mean (SD)].

	Group		*p* value
	Schizotypy (n = 20)	Non-schizotypy (n = 24)	
**Certain go trials**			
Accuracy of trials (%)	93.85 (3.91)	93.33 (4.00)	0.651
RT of correct trials (ms)	391.78 (49.79)	420.42 (60.95)	0.100
**17% uncertain go trials**			
Accuracy of trials (%)	96.45 (2.65)	94.96 (6.35)	0.303
RT of correct trials (ms)	440.30 (73.04)	547.14 (108.39)	0.001
**33% uncertain go trials**			
Accuracy of trials (%)	96.15 (3.36)	90.71 (10.02)	0.018
RT of correct trials (ms)	479.80 (87.60)	592.57 (118.12)	0.001
**17% stop trials**			
Accuracy of trials (%)	48.15 (2.94)	52.17 (4.42)	0.001
SSRT	219.98 (26.84)	191.05 (45.92)	0.017
**33% stop trials**			
Accuracy of trials (%)	50.60 (3.46)	54.29 (5.84)	0.013
SSRT	192.24 (35.57)	162.28 (56.12)	0.045

*RT* response time, *SSRT* stop signal reaction time.

#### Reactive inhibition (stop trials)

**Figure 2:**
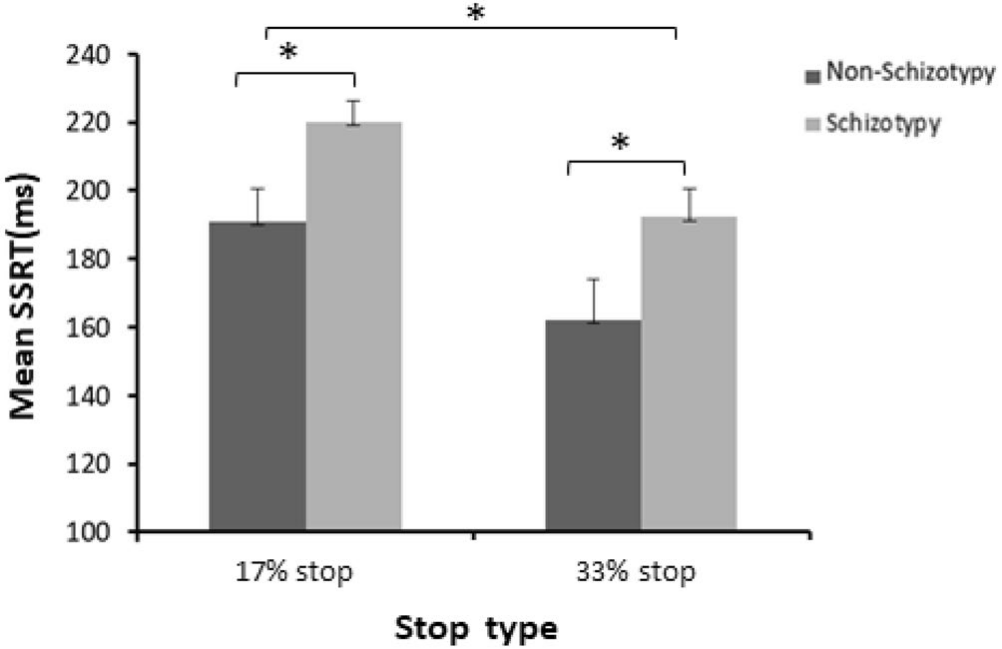
Stop-signal reaction time (SSRT) of “17% stop” trials and “33% stop” trials for the non-schizotypy and schizotypy group. Error bars represent standard error. *p < .05.

There was a significant main effect of Stop type (*F*_(1, 42)_ = 18.59, *p* < 0.001, *η*_*p*_^2^ = 0.307), the “17% stop” trials showed longer SSRT (*M* = 205 ms) than “33% stop” trials (*M* = 177 ms) (Table 1). The main effect of Group was also significant (*F*_(1, 42)_ = 6.67, *p* = 0.013, *η*_*p*_^2^ = 0.137) suggesting that the schizotypy group exhibited longer SSRT (*M* = 206 ms) compared with the non-schizotypy group (*M* = 177 ms). The Group × Stop type interaction was not significant (*F*_(1, 42)_ = 0.006, *p* = 0.938, *η*_*p*_^2^ = 0.001) (Fig. 2).

### ERP results

The number of valid trials in each condition to extract ERP components for each group are presented as follows:

For non-schizotypy group, the min, max, and mean (± SD) number of valid trials for “certain go” trials were 40, 59, and 52.83 (± 5.10) respectively; for “17% uncertain go” trials were 250, 298, and 283.71 (± 13.41) respectively; for “33% uncertain go” trials were 97, 120, and 113.04 (± 6.64) respectively; for successful “17% stop” trials were 26, 36, and 29.96 (± 2.42) respectively; for failed “17% stop” trials were 22, 31, and 27.46 (± 2.70) respectively; for successful “33% stop” trials were 26, 40, and 31.08 (± 3.16) respectively; for failed “33% stop” trials were 19, 31, and 26.71 (± 3.50) respectively.

For schizotypy group, the min, max, and mean (± SD) number of valid trials for “certain go” trials were 42, 58, and 52.70 (± 4.77) respectively; for “17% uncertain go” trials were 206, 297, and 280.25 (± 21.64) respectively; for “33% uncertain go” trials were 83, 120, and 111.75 (± 9.94) respectively; for successful “17% stop” trials were 22, 32, and 28.30 (± 2.18) respectively; for failed “17% stop” trials were 15, 37, and 29.65 (± 3.91) respectively; for successful “33% stop” trials were 23, 35, and 29.50 (± 2.86) respectively; for failed “33% stop” trials were 23, 31, and 28.40 ± 2.48 respectively.

#### Proactive inhibition (go trials)

##### N1 amplitude

**Figure 3:**
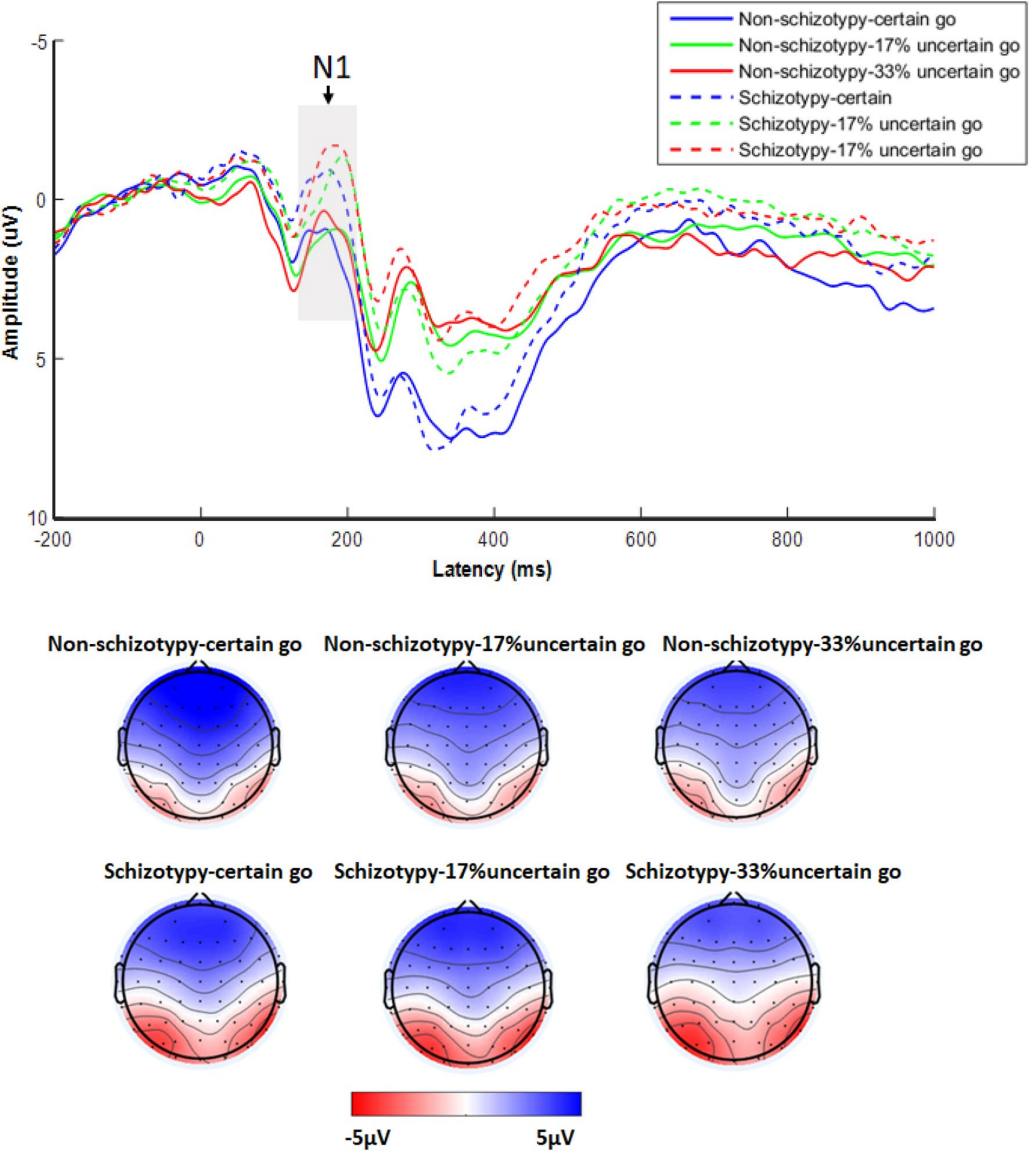
Grand-average event-related potentials (ERPs) elicited by different Go type at the Pz, P1, and P2 recording site for the non-schizotypy and schizotypy group. The gray-shaded areas indicate the 140- to 210-ms time window for the calculation of the mean value of the N1 wave. The time point “0” indicates stimuli onset.

There was a significant main effect of Group (*F*_(1, 42)_ = 7.42, *p* = 0.009, *η*_*p*_^2^ = 0.150), suggesting that the N1 was more negative for the schizotypy than the non-schizotypy group. The main effect of Go type was also significant (*F*_(2, 84)_ = 4.96, *p* = 0.016, *η*_*p*_^2^ = 0.106). Subsequent post-hoc tests with Bonferroni correction indicated that the N1 amplitude induced by “33% uncertain go” condition was more negative than that in the “17% uncertain go” (*p* = 0.031) and “certain go” (*p* = 0.041) conditions. However, the difference of N1 amplitude between “17% uncertain go” and “certain go” conditions was not significant (*p* = 0.752). There was no interaction between Group and Go type (*F*_(2, 84)_ = 0.72, *p* = 0.458, *η*_*p*_^2^ = 0.017) (Fig. 3).

##### P3 amplitude

**Figure 4:**
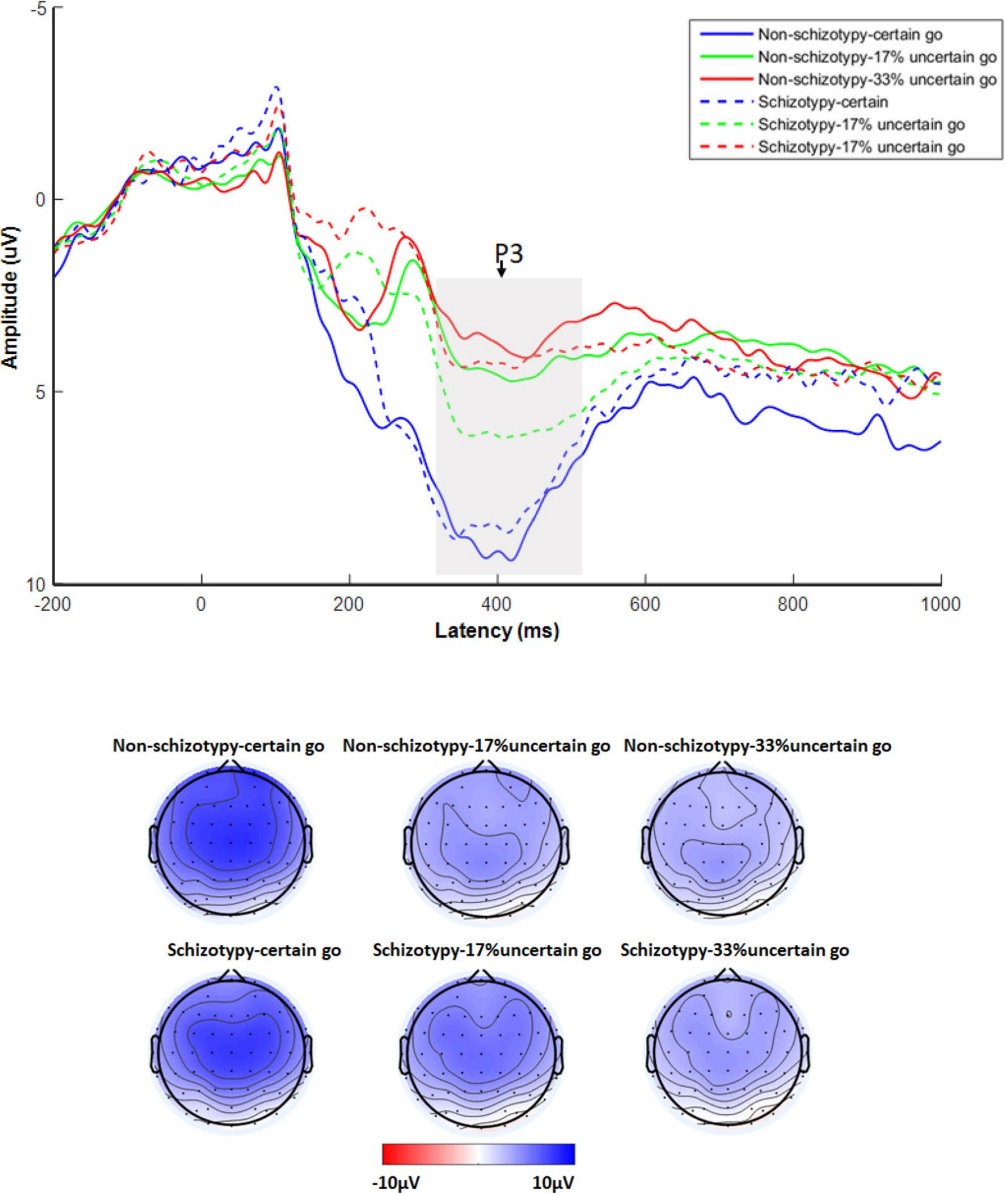
Grand-average event-related potentials (ERPs) elicited by different Go type at the Cz, C1, and C2 recording site for the non-schizotypy and schizotypy group. The gray-shaded areas indicate the 320- to 520-ms time window for the calculation of the mean value of the P3 wave. The time point “0” indicates stimuli onset.

There was a significant main effect of Go type (*F*_(2, 84)_ = 81.52, *p* < 0.001, *η*_*p*_^2^ = 0.660). Subsequent post-hoc tests with Bonferroni correction indicated that the P3 amplitude for the “certain go” trials was more positive than that for the “17% uncertain go” trials (*p* < 0.001), which was more positive than that for the “33% uncertain go” trials (*p* < 0.001). The main effect of Group was not significant (*F*_(1, 42)_ = 1.024, *p* = 0.317, *η*_*p*_^2^ = 0.024). However, there was a significant Group × Go type interaction (*F*_(2, 84)_ = 4.14, *p* = 0.033, *η*_*p*_^2^ = 0.090), simple effect analysis revealed that for the "17% uncertain go" trials, schizotypy individuals showed significantly larger P3 amplitude than HC (*F*_(1, 42)_ = 3.93, *p* = 0.050, *η*_*p*_^2^ = 0.085). However, this was not the case for the "certain go" (*F*_(1, 42)_ = 0.007, *p* = 0.934, *η*_*p*_^2^ = 0.000) or "33% uncertain go" trials (*F*_(1, 42)_ = 0.66, *p* = 0.421, *η*_*p*_^2^ = 0.015) (see Fig. 4). In order to avoid the potential confounding caused by different groups on brain architecture and morphological factors like skull thickness in EEG data, it is better to do within group comparisons between conditions, thus we also conducted two 2 × 2 ANOVAs. For the first 2 (Group: schizotypy, non-schizotypy) × 2 (Go type: certain go, 17% uncertain go) ANOVA, the interaction was significant, *F*_(1,42)_ = 6.57, *p* = 0.014, *η*_p_^2^ = 0.135. For the non-schizotypy group, the P3 amplitude difference between “17% uncertain go” trials and “certain go” trials was significant (*p* < 0.001); for the schizotypy group, the difference was also significant (*p* = 0.001); however, the difference between different type of trials was larger in the non-schizotypy group than in the schizotypy group, suggesting larger proactive inhibition in the non-schizotypy group in the 17% stop condition. However, for the second 2 (Group: schizotypy, non-schizotypy) × 2 (Go type: certain go, 33% uncertain go) ANOVA, the interaction was not significant, *F*(1,42) = 0.80, *p* = 0.377, *η*_p_^2^ = 0.0019, suggesting no significant group difference on proactive inhibition in the 33% stop condition.

#### Reactive inhibition (stop trials)

##### N2 amplitude (“17% stop” trials)

**Figure 5:**
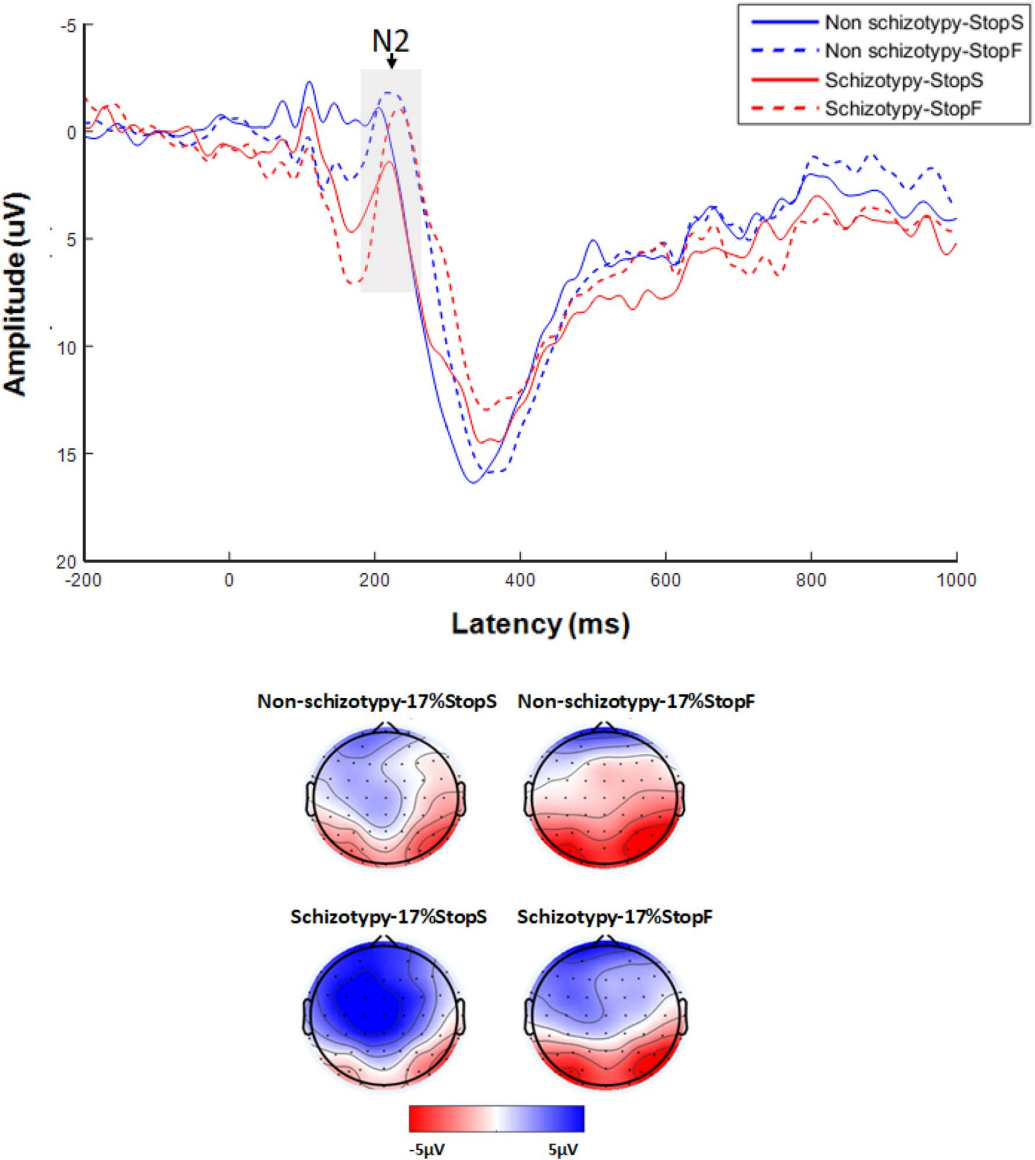
Grand-average event-related potentials (ERPs) elicited by “17% stop” trials associated with successful stop (StopS) and failed stop (StopF) at the Fz, F1, and F2 recording site for the non-schizotypy and schizotypy group. The gray-shaded areas indicate the 175- to 250-ms time window for the calculation of the mean value of the N2 wave. The time point “0” indicates stimuli onset.

There was a marginal significant main effect of Stop response (*F*_(1, 42)_ = 3.89, *p *= 0.055, *η*_*p*_^2^ = 0.085), suggesting that the N2 tended to be more negative for failed than successful stop trials. The main effect of Group was significant (*F*_(1, 42)_ = 4.39, *p *= 0.042, *η*_*p*_^2^ = 0.095), indicating smaller N2 amplitude in individuals with schizotypy. The interaction between Group and Stop response was not significant (*F*_(1, 42)_ = 0.439, *p *= 0.511, *η*_*p*_^2^ = 0.010) (Fig. 5).

##### N2 amplitude (“33% stop” trials)

There was a significant main effect of Stop response (*F*_(1, 42)_ = 10.775, *p* = 0.002, *η*_*p*_^2^ = 0.204), suggesting that the N2 was more negative for failed than successful stop trials. The main effect of Group (*F*_(1, 42)_ = 0.147, *p* = 0.703, *η*_*p*_^2^ = 0.003) and interaction between Group and Stop response (*F*_(1, 42)_ = 0.001, *p* = 0.973, *η*_*p*_^2^ = 0.001) were not significant.

### Correlation analyses

First, we calculated the behavioural preparatory processing indexes: 17% preparatory processing (response time difference between “17% uncertain go” and “certain go” trials) and 33% preparatory processing (response time difference between “33% uncertain go” and “certain go” trials); then, we calculated the N1 amplitude difference in 17% stop condition (N1 amplitude difference between “17% uncertain go” and “certain go” trials) and 33% stop condition (N1 amplitude difference between “33% uncertain go” and “certain go” trials) respectively; similar P3 amplitude differences were also calculated.

Then, relevant correlation analyses were conducted. Results indicated that the 17% N1 amplitude difference and 17% preparatory processing was not significantly correlated (*r* = − 0.15, *p* = 0.320), the 33% N1 amplitude difference and 33% preparatory processing was not significantly correlated either (*r* = 0.05, *p* = 0.753). The above results indicated that the N1 was not related to proactive inhibition.

In addition, 17% P3 amplitude difference and 17% preparatory processing was negatively correlated (*r* = − 0.72, *p* < 0.001), 33% P3 amplitude difference and 33% preparatory processing was also negatively correlated (*r* = − 0.57, *p* < 0.001). The above results indicated that the P3 was associated with proactive inhibition. Results of correlation analyses in each group were generally the same as the results of the combined group and are presented in Supplementary Information.

## Discussion

To our knowledge, this is the first study to investigate the neural correlates of proactive inhibition (i.e., anticipation of stopping) and reactive inhibition (i.e., outright stopping) in individuals with schizotypy. There are two main findings. First, individuals with schizotypy exhibited impairments in proactive response inhibition, manifested in a smaller increase in response time of go trials as the probability of stop signal increasing. At the neural level, we found increased P3 for go trials in the 17% stop condition in schizotypy compared with non-schizotypy individuals. Second, individuals with schizotypy exhibited impairments in reactive response inhibition, manifested in longer SSRT in both 17% and 33% stop conditions and reduced N2 amplitude for stop trials in the 17% stop condition.

Both schizotypy and non-schizotypy individuals exhibited increased response time for go trials as the probability of stop signal increasing, these findings were consistent with previous studies^17,21–23^. However, there was a smaller increase in response time on both “17% uncertain go” and “33% uncertain go” trials in schizotypy compared to non-schizotypy individuals, suggesting reduced proactive inhibition in schizotypy individuals. This is consistent with a previous study in schizophrenia patients and their unaffected siblings^26^. All these findings suggested that a lesser response time increase with the probability of stop-signal increasing maybe present in the schizophrenia spectrum, indicating poor proactive inhibition in these individuals. It should be noted that increased response time for go trials in non-schizotypy individuals also led to a higher omission error rate for the “33% uncertain go” trials than individuals with schizotypy. This is a manifestation of their higher anticipation of stop signals. It should be noted that there is an alternative explanation for the behavioural results: It is a tradeoff of allocating attention resources between “go” task and “stop” task. For the schizotypy group, they allocated more resources to the “go” task, thus they showed higher accuracy and shorter RT on uncertain go trials but performed worse on stop trials (lower rate of successful stop and longer SSRT); for the non-schizotypy group, they allocated more resources to the “stop” task, thus they showed lower accuracy and longer RT on uncertain go trials but performed better on stop trials (higher rate of successful stop and shorter SSRT). It is consistent with previous studies that healthy controls responded more cautiously compared with schizophrenia patients when there was chance of having to inhibit their response, which was accompanied by longer reaction time to uncertain go trials^13,26^. However, it is still a problem for individuals with schizotypy not using the context information to guide their behaviour, i.e., when stop signals would appear, they still focused on the “go” task. This is consistent with a previous viewpoint that individuals with schizotypy had context integration deficits^48^. Further studies are needed to examine the alternative explanations.

At the neural level, individuals with schizotypy showed increased N1 amplitude on “go” trials compared with non-schizotypy individuals. N1 reflects sensory processing and stimulus discrimination^38,39^. In the present study, we did not find significant correlations between N1 amplitude and preparatory processing in the 17% or 33% condition, indicating that N1 may not be involved in the proactive control process. Previous studies suggested that N1 amplitude is larger when a stimulus was attended than when it was ignored^40,49^. It is possible that compared with non-schizotypy individuals, schizotypy individuals tend to allocate more attention resources to discriminate the feature of the go stimuli (i.e., circle or triangles) in order to get a high accuracy in the Go task, which induced a larger N1 amplitude. This may lead to less attention resource on preparation for stop signal, which indirectly lead to reduced proactive inhibition in individuals with schizotypy. The present finding that individuals with schizotypy exhibited increased N1 amplitude is consistent with previous studies showing positive schizotypy associated with increased N1 amplitude^50,51^. However, several studies demonstrated that positive schizotypy was associated with reduced N1 amplitude^52,53^. These inconsistencies might be related to different experimental tasks, different measures of schizotypy, and different schizotypy score used (a specific dimension score vs. total score) in these studies^52,54^. At least, they all suggested that schizotypy was associated with abnormal N1 amplitude.

Our findings showed that the P3 amplitude of “go” trials reduced as the probability of stop signal increasing, while response time of “go” trials showed a reversed pattern, i.e., “33% uncertain go” trials showed smaller P3 amplitude and longer response time relative to “17% uncertain go” trials, which showed smaller P3 amplitude and longer response time compare with “certain go” trials. This is consistent with a study using the stop signal task and found that slower responses to go trials induced smaller P3 amplitude relative to faster responses^2^. Taken together, we may consider P3 reflecting proactive inhibition in the present study, i.e., the preparation for suppressing the responses in stop trials. This is also supported by the results of correlational analyses, which showed that greater preparatory processing (larger response time difference between uncertain go and certain go trials) was correlated with smaller P3 amplitude. That means the higher the proactive inhibition, the smaller the P3 amplitude on “go” trials. In the present study, smaller P3 amplitude may indicate more preparation for the stop signal, i.e., stronger proactive inhibition.

Moreover, we observed that schizotypy individuals exhibited larger P3 amplitude on “go” trials in the 17% stop condition compared with non-schizotypy individuals, suggesting reduced proactive inhibition. This finding provided, for the first time, insights into mechanisms underlying reduced proactive inhibition in schizotypy individuals. A previous study using functional magnetic resonance imaging found reduced activation of right striatum, the right inferior frontal cortex, and bilateral temporoparietal junction during proactive inhibition in schizophrenia patients and their unaffected siblings compared with healthy control individuals^26^. Taken together, these results suggest that individuals at risk for schizophrenia presented impaired neural responses of proactive inhibition.

Individuals with schizotypy exhibited longer SSRT compared to non-schizotypy individuals at the behavioural level, suggesting impaired reactive inhibition. This finding is consistent with previous studies in schizotypy individuals^4,5^. Studies have also shown that longer SSRT was presented in patients with schizophrenia^47,55^ and children at genetic risk for schizophrenia^14^, supporting the view that longer SSRT is one of the biological markers for schizophrenia. It should be noted that there was no significant interaction between Group and Stop type, i.e., individuals with schizotypy were impaired in 17% and 33% stop conditions to a similar degree. The finding indicates that the impairment of reactive inhibition in schizotypy individuals did not vary with the probability of stop trials. At the neural level, N2 component was observed in stop trials, and the amplitude of N2 was larger for “failed stop” than “successful stop” trials, which is consistent with previous studies^2,46^, suggesting that N2 is associated with the evaluation of the stop-signal and detection of a stop failure^2,43,47^. More importantly, we found that schizotypy individuals exhibited a significant reduction of N2 amplitude on the “17% stop” trials (both successful and failed) compared with the non-schizotypy individuals, suggesting reduced ability to evaluate and detect stop signals in schizotypy. The present study provides evidence for reactive inhibition impairment in schizotypy individuals for the first time at the neural level. A previous study demonstrated that schizophrenia patients showed reduced N2 amplitude on stop trials and longer SSRT^47^. The present study suggested that impaired reactive inhibition in patients with schizophrenia may not be caused by medication or chronicity.

It should be noted that in the “33% stop” condition, there was no significant difference between schizotypy and non-schizotypy individuals on P3 amplitude for go trials, indicating relatively intact proactive inhibition of schizotypy individuals in this condition. The two groups of participants did not show significant difference on N2 amplitude for stop trials in the “33% stop” condition either. One possible explanation is that the relatively frequent stop signals in the 33% stop condition could prompt schizotypy individuals making sufficient preparation or anticipation to withhold responses in stop trials as non-schizotypy individuals at the neural level, with this preparation, both proactive and reactive response inhibition showed no group difference. However, further studies are needed to examine this issue.

The present study has several limitations. First, we only manipulated three stop-signal probability levels, a more refined delineation of stop-signal probability levels is needed in future studies to examine more precisely how proactive and reactive inhibition vary with contexts. Second, this study examined proactive and reactive response inhibition with ERP technique in individuals with schizotypy, recruiting participants with high positive and high negative schizotypy separately would make it clear which dimension of schizotypy contributed to the current findings; moreover, recruiting clinical sample of schizophrenia could examine whether similar results would be exhibited in patients with schizophrenia. Third, individuals with schizotypy exhibited shorter response time than non-schizotypy individuals, this might be related to higher impulsivity in schizotypy. However, we did not include measures on impulsivity in this study, future studies are needed to exclude the potential confounding of impulsivity.

Notwithstanding the above limitations, the present study suggests that the response inhibition impairment in schizotypy is not only accompanied by deficits in outright stopping (reactive inhibition) but also impaired preparation for stopping (proactive inhibition). Our results contribute to a better understanding of the neural mechanisms underlying proactive (increased P3 amplitude for go trials in the 17% stop condition) and reactive inhibition (decreased N2 amplitude for stop trials in the 17% stop condition) in schizotypy individuals.

## Methods

### Participants

Five-hundred university students from Beijing were recruited and completed the Schizotypal Personality Questionnaire (SPQ)^56,57^ online. Participants who obtained a total score within the top 10% on the SPQ were considered as individuals with schizotypy, whereas participants who scored below average were considered as non-schizotypy group according to the manual of SPQ^57^. Exclusion criteria were: (1) a current diagnosis or a history of neurological or psychiatric disorders; (2) a first-degree relative with psychiatric disorders; (3) alcohol/substance abuse/dependence; (4) colour blindness. A total of 56 participants (26 schizotypy and 30 non-schizotypy) were recruited to take part for the ERP experiment. Seven participants (4 schizotypy individuals and 3 non-schizotypy) with frequent blink artifacts or muscular artifacts were excluded from the subsequent analysis. Five participants (2 schizotypy individuals and 3 non-schizotypy) were excluded for not following the task instructions and deliberately waited for the stop signal or failing to stop on more than 80% of stop trials. Finally, a total of 44 participants were included in the final analysis. The ethics committee of the Institute of Psychology, Chinese Academy of Sciences approved this study. All methods were carried out in accordance with relevant guidelines and regulations. Written informed consent was obtained from all participants. Participants were paid after finishing the study.

### Task and procedure

**Figure 6:**
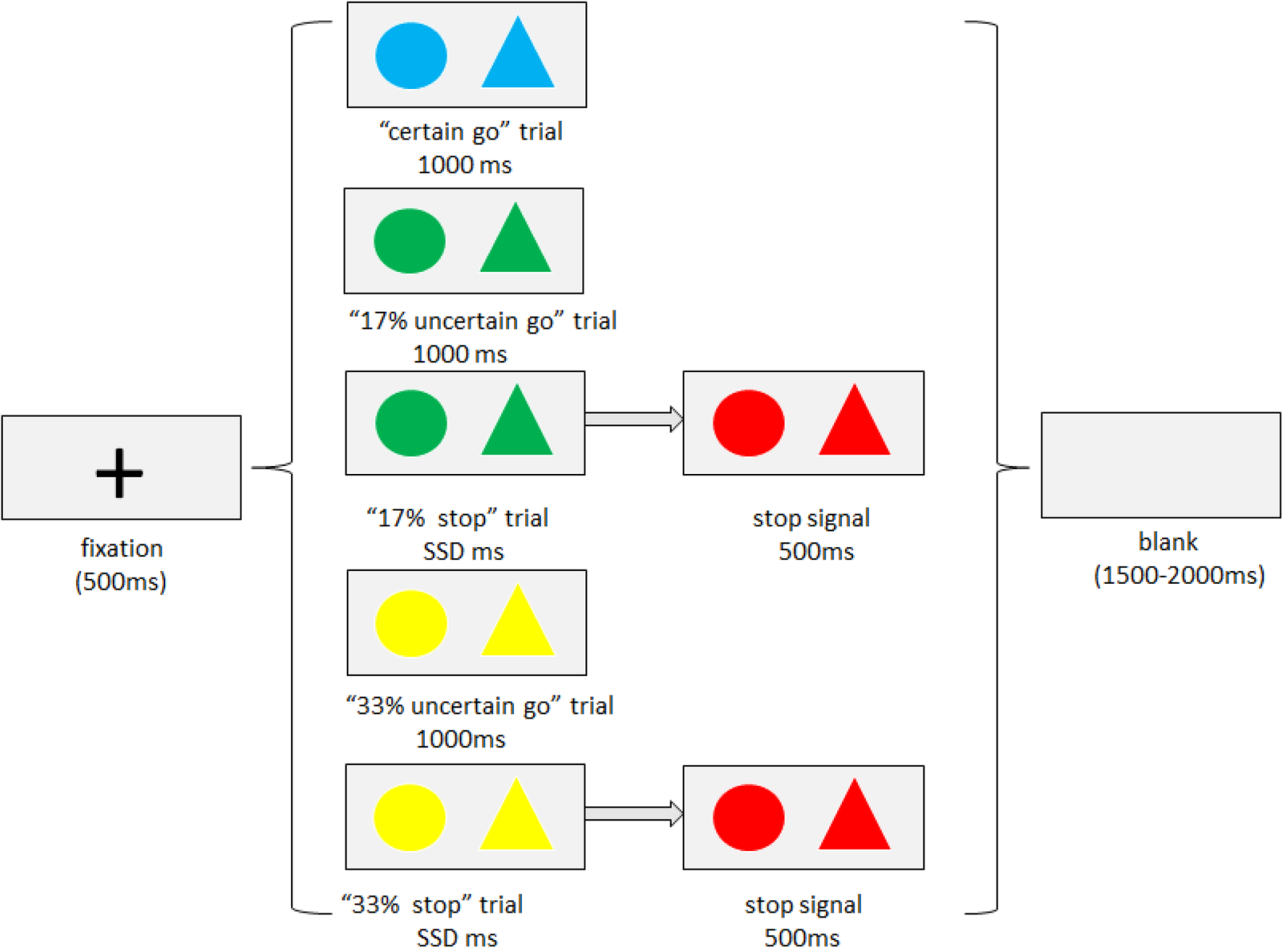
The adapted stop-signal task for the present study. 0%, 17% and 33% stop conditions appeared in a block form and the order of conditions was counterbalanced across sessions. In the 0% condition, only “certain go” trials are presented, the color of stimuli is blue. Each trial started with a black fixation presented for 500 ms, followed by a blue circle or triangle presented for 1000 ms, then a blank presented with randomly duration between 1500 and 2000 ms. Participants were instructed to press the “J” key in response to circles and “F” key to triangles as fast as possible. In the 17% stop condition, both “go” trials and “stop” trials are presented, the color of go stimuli is green, participants were instructed to make response to circles and triangles as 0% stop condition (“17% uncertain go” trials), however, for 17% probability, the color changed to red after a short delay (SSD), and subjects were required to withhold the response (“17% stop” trials). The SSD started was initially at 250 ms and varied from one stop trial to the next based on a staircase procedure. The 33% stop condition was the same as the 17% stop condition, except for the color of go stimuli (“33% uncertain go” trials) is yellow and the probability that a stop-signal would occur was 33%.

We used an adapted stop-signal task^10^ in this study. The modified version measures both proactive and reactive response inhibition. It consisted of three conditions based on the probability of stop signal: 0% (no stop trials), 17% (17% stop trials) and 33% (33% stop trials) (Fig. 6).

For the 0% stop condition, only “go” trials were presented, the stimuli were blue in color. Each trial started with a black fixation at the center of a grey background for 500 ms, followed by a blue circle or triangle presented for 1000 ms, participants were instructed to press the “J” key in response to circles and “F” key in response to triangles as fast as possible, and then a blank screen (the duration varied randomly between 1500 and 2000 ms). The 17% stop condition contained “go” trials and “stop” trials, the probability of “stop” trials was 17%, these trials were pseudorandomly interspersed between “go” trials, ensuring that the first trial of each stop condition was not a stop trial and no consecutive stop trials occurred. In this condition, the color of stimuli was green, the presentation sequence of each “go” trial was the same as the 0% stop condition. For the “stop” trials, the color of stimuli would turn red after a short delay (Stop-signal delay, SSD), which indicated that participants need to withhold their response. The SSD started at 250 ms and varied from one “stop” trial to the next based on a tracking procedure (Band et al., 2003): if the participant succeeded in withholding the response, the SSD increased by 50 ms for the next “stop” trial, making it more difficult to stop; if the participant failed, the SSD decreased by 50 ms for the next “stop” trial. With this tracking procedure, the accuracy for the “stop” trials was kept around 50%. The 33% stop condition was the same as the 17% stop condition, except for the color of the “go” stimuli was yellow and the probability of the “stop” trial was 33%.

Trials were presented in 6 sessions, each session included all three conditions and consisted of 100 trials (0% stop condition: 10 “go” trials; 17% stop condition: 50 “go” trials and 10 “stop” trials; 33% stop condition: 20 “go” trials and 10 “stop” trials). In each session, the trials in each condition appeared in a block form, i.e., after all trials in one condition presented, then the trials of another condition presented. The order of conditions was counterbalanced across sessions. In total, there were 60 “go” trials in the 0% stop condition, 300 “go” trials and 60 “stop” trials in the 17% stop condition, and 120 “go” trials and 60 “stop” trials in the 33% stop condition.

In 17% and 33% stop conditions, participants may respond slower in “go” trials (i.e. anticipation or preparation) than in the 0% stop condition in order to withhold response in “stop” trials^22^. Therefore, the difference in response time between the “go” trials in the 0% stop condition and the “go” trials in the 17% or 33% stop condition was considered as the preparatory processing. All response time were calculated based on trials with correct responses. The reactive inhibition was estimated with the SSRT which was calculated for 17% and 33% stop conditions separately, using the integration method^58^. With this method, the SSRT was calculated with the following steps: (1) calculate the number (n) of go trials with correct responses; (2) order the response times of these go trials with correct responses; (3) calculate the number (a), a = n × proportion of failed stop for stop trials; (4) select the *a*th response time list from step (2); (5) SSRT = *a*th response time (from step 4) − mean SSD.

In order to distinguish “go” trials and “stop” trials in different conditions and make subsequent data analysis more convenient, we named “go” trials in 0%, 17% and 33% stop conditions as “certain go” trials, “17% uncertain go” trials and “33% uncertain go” trials, respectively. Similarly, the “stop” trials in 17% and 33% stop conditions were named as “17% stop” trials and “33% stop” trials, respectively.

Before the beginning of formal experiment, participants completed a short practice session to ensure they understand the task. After the practice session, they completed six experimental sessions with short breaks between sessions. Participants were emphasized that speed and accuracy of “go” trials were of equal importance as “stop” trials and it would be impossible to successfully suppress all responses when a stop-signal occurred. Participants were told that stop-signals would never appear when the stimuli were presented in blue and that the probability of stop trials was smaller when stimuli were presented in green than in yellow.

### Electrophysiological recording and preprocessing

Continuous EEG was recorded using a 64-channel elastic cap with an online left mastoid reference. Vertical and horizontal electrooculogram (EOG) were recorded from electrodes placed below the left eye and the outer canthi of both eyes, respectively. All electrodes impedance was kept below 5kΩ. EEG and EOG signals were sampled at 1000 Hz (Neuroscan system, Scan 4.5) and amplified by a 0.05–100 Hz online band pass filter.

Off-line analysis was performed using the EEG-lab Toolbox. Firstly, all EEG signals were off-line re-referenced to the average of left and right mastoids. Then these signals were filtered with a 0.1–30 Hz band-pass digital filter. The EEG signals were segmented into epochs using 200 ms pre-stimulus onset until 1000 ms poststimulus. Each segment was baseline corrected to the 200 ms pre-stimulus time window. Independent Component Analysis (ICA) was implemented in EEGLAB for the removal of blink artifacts, trials that exceeded a threshold of ± 100 uv were excluded. Visual inspection was also conducted to exclude abnormal EEG data before and after ICA. The remaining trials after the above data preprocessing were considered to be artifact-free, then the separate averages for different trial types were computed. From visual inspection of the grand average data, N1 and P3 components for go trials were analyzed. According to the distribution of the N1 and P3 in previous studies^37,38,47^ and in the current study, three electrodes in the parietal region (P1, P2, PZ) and three electrodes in the central region (C1, C2, CZ) were selected for analysis on the amplitude of N1 and P3, respectively. The time window for N1 was 140–210 ms and for P3 was 320–520 ms after stimulus onset. The N2 for stop trials was considered to be associated with reactive response inhibition, and was measured during 175–250 ms after stop signal onset at electrodes Fz, F1 and F2. Voltages were averaged across selected electrodes, in order to increase the signal-to-noise ratio and simplify the statistical models^59^. The mean amplitude was used for all analyses.

### Statistical analysis

To evaluate whether the two groups differ in demographic variables such as age, gender ratio (female:male), and years of education, independent samples *t* test or χ^2^ were used.

Behavioural data: for proactive response inhibition, a 2 (Group: schizotypy vs. non-schizotypy) × 3 (Go type: “certain go” vs. “17% uncertain go” vs. “33% uncertain go”) mixed analysis of variance (ANOVA) was carried out on response time for correct go trials; for reactive response inhibition, a 2 (Group: schizotypy vs. non-schizotypy) × 2 (Stop type: “17% stop” vs. “33% stop”) ANOVA was carried out on SSRT.

ERP data: for proactive inhibition, a 2 (Group: schizotypy vs. non-schizotypy) × 3 (Go type: “certain go” vs. “17% uncertain go” vs. “33% uncertain go”) mixed ANOVA was carried out on N1 and P3 amplitude; for reactive inhibition, a 2 (Group: schizotypy vs. non-schizotypy) × 2 (Stop response: successful stop vs. failed stop) mixed ANOVA on N2 amplitude was carried out for “17% stop” and “33% stop” trials separately.

In order to examine whether ERP components of “uncertain go” trials represent proactive inhibition, we conducted correlation analyses between ERP components (N1 and P3 amplitude difference between uncertain go and certain go trials) and preparatory processing (response time difference between uncertain go and certain go trials) in 17% and 33% stop conditions separately, the correlation analyses were based on data from all participants. For all analyses, the significance level was set at 0.05.

## Supplementary Information

Supplementary Information.

## Data Availability

The datasets generated during and/or analysed during the current study are available from the corresponding author upon reasonable request.
