# Supplementary material for: An ERP study on proactive and reactive response inhibition in individuals with schizotypy
Source: Sci Rep. 2021 Apr 16;11:8394. doi: 10.1038/s41598-021-87735-5 (PMC8052443; doi:10.1038/s41598-021-87735-5)
Supplement: Supplementary file 1 — Supplementary Information. [file 41598_2021_87735_MOESM1_ESM.pdf]

An ERP study on proactive and reactive response inhibition in individuals with schizotypy

Lu-xia Jia[1,2], Xiao-jing Qin[1,2], Ji-fang Cui[3], Qi Zheng[1,2], Tian-xiao Yang[1,2], Ya Wang[1,2*], Raymond C. K. Chan[1,2]

[1] Neuropsychology and Applied Cognitive Neuroscience Laboratory, CAS Key Laboratory of Mental Health, Institute of Psychology, Beijing, China

[2] Department of Psychology, University of Chinese Academy of Sciences, Beijing, China

[3] Research Center for Information and Statistics, National Institute of Education Sciences, Beijing, China

*Email: wangyazsu@gmail.com, wangya@psych.ac.cn

Supplementary materials

Results of correlation analyses between behavioural preparatory processing and ERP data in each group

First, we calculated the behavioural preparatory processing indexes: 17% preparatory processing (response time difference between “17% uncertain go” and “certain go” trials) and 33% preparatory processing (response time difference between “33% uncertain go” and “certain go” trials); then, we calculated the N1 amplitude difference in 17% stop condition (N1 amplitude difference between “17% uncertain go” and “certain go” trials) and 33% stop condition (N1 amplitude difference between “33% uncertain go” and “certain go” trials) respectively; similar P3 amplitude

differences were also calculated. Then, relevant correlation analyses were conducted.

For the N1 amplitude

In the non-schizotypy group:

The 17% preparatory processing and N1 amplitude difference was not significantly correlated (r=-0.0001, p=0.998), the 33% preparatory processing and N1 amplitude difference was not significantly correlated either (r=0.19, p=0.385).

In the schizotypy group:

The 17% preparatory processing and N1 amplitude difference was not significantly correlated (r=-0.26, p=0.261), the 33% preparatory processing (response time difference "between 33% uncertain" and "certain go" trials) and N1 amplitude difference was not significantly correlated either (r=-0.13, p=0.597).

From the above results, we found that consistent with the results of combined group, no significant correlations between preparatory processing and N1 amplitude difference were observed in either group.

For the P3 amplitude

In the non-schizotypy group:

The 17% preparatory processing and P3 amplitude difference was significantly correlated (r=-0.71, p<0.001), the 33% preparatory processing and P3 amplitude difference was also significantly correlated (r=-0.64, p<0.001).

In the schizotypy group:

The 17% preparatory processing and P3 amplitude difference was significantly correlated (r=-0.56, p=0.011), the 33% preparatory processing and P3 amplitude difference showed a trend to be significantly correlated (r=-0.40, p=0.085).

The above results indicated that generally consistent with the results of combined group, the correlations between P3 amplitude difference and preparatory processing were generally significant in both the non-schizotypy and schizotypy groups. From the following figures, we can also see that the correlations showed a similar pattern in the two groups.

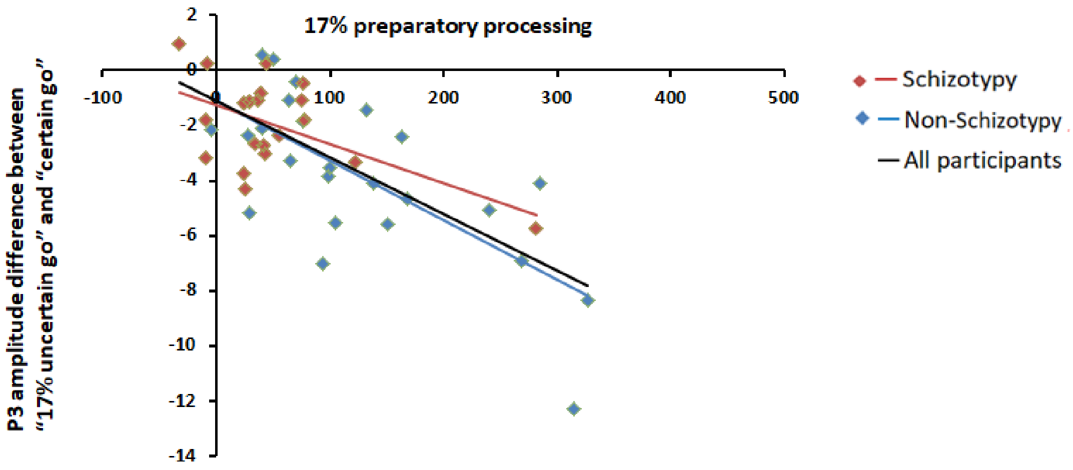

Fig. S1 Correlation between 17% preparatory processing and P3 amplitude difference

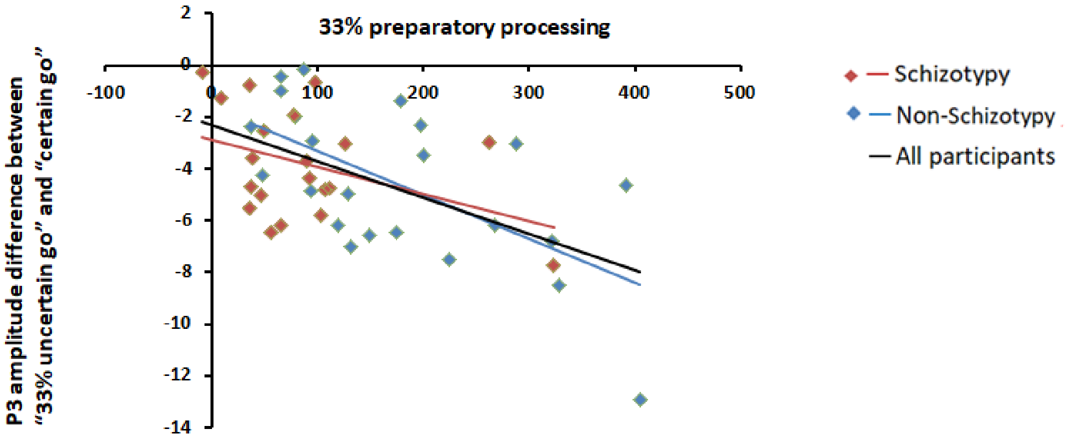

Fig. S2 Correlation between 33% preparatory processing and P3 amplitude difference
